# Preparation of soy sauce by walnut meal fermentation: Composition, antioxidant properties, and angiotensin‐converting enzyme inhibitory activities

**DOI:** 10.1002/fsn3.1453

**Published:** 2020-02-17

**Authors:** Jie Xu, Feng Jin, Jing Hao, Joe M. Regenstein, Fengjun Wang

**Affiliations:** ^1^ Beijing Key Laboratory of Forest Food Processing and Safety Department of Food Science and Engineering College of Biological Sciences and Biotechnology Beijing Forestry University Beijing China; ^2^ Department of Food Science Cornell University Ithaca NY USA

**Keywords:** ACE inhibitory activity, amino acid, antioxidant activity, soy sauce, walnut

## Abstract

Remaining walnut meal after oil extraction still contains many nutrients. However, these by‐products have not been effectively used. In this study, walnut meal and *Aspergillus oryzae* (3.042) were used in combination to prepare a soy sauce‐like material with high amino nitrogen content (ANC). The optimal conditions for the preparation of walnut soy sauce (ratio of brine:koji of 1.7:1 for 6 days at 45°C) were determined using response surface experiments (RSE), which showed maximum ANC of 855 mg/100 ml. The results of amino acid analysis indicated that walnut soy sauce had a similar amino acid composition compared with three commercial soy sauces. It contained all the essential amino acids and had a high content of umami amino acids such as Glu and Asp, which may give it a stronger umami taste. Moreover, the crude walnut soy sauce was extracted sequentially using ethyl acetate, n‐butanol, acetone and water, and the total phenols, total flavonoids, reducing sugars, and the peptides of different solvent extracts were measured. Results showed that the total phenolic and flavonoid contents were highest in the ethyl acetate extracts. However, water residue had the highest levels of reducing sugars and peptides. In vitro, the water residue showed the highest antioxidant capacity and angiotensin‐converting enzyme (ACE) inhibitory activity, due to more reducing sugars and peptides. These results indicated that walnut soy sauce may have significant antioxidant and ACE inhibitory activity. The findings provide a scientific basis for developing a replacement for soy sauce and broaden the beneficial application of walnut meal.

## Graphical abstract

The optimal conditions for the preparation of walnut soy sauce were determined. Walnut soy sauce has high umami amino acid content. All four extracts had antioxidant and ACE inhibitory activities.

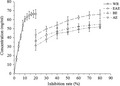

## 1 INTRODUCTION

Soy sauce is a seasoning and coloring agent widely used in food preparation in Asian countries, especially China, Japan, and Korea (Liu et al., 2015). The product is not only nutritious, but also contains a variety of physiologically active ingredients, such as furanone, phenolic acids, organic acids, and peptides (Li, Zhao, Su, Lin, & Wang, 2016). Their presence provides soy sauce some properties, such as antioxidant, anti‐cancer, lowering high uric acid, and slowing the progression of cataracts (Kataoka, 2005). Due to its rich flavor and unique aroma, the use of soy sauce in western countries has been increasing (Steinhaus & Schieberle, 2007).

Soy sauce is traditionally produced by fermenting a combination of soybeans and wheat grains in brine (Steinhaus & Schieberle, 2007). It can be divided into sweet, salty, dark, and light soy sauces (Yamamoto, Shiga, Kodama, Imamura, Uchida, & Obata, 2014) according to the added ingredients, for example, sugar and salt, and processing methods (Jakobi, Salmen, Paulus, Tolan, & Rosenhahn, 2017). Recent research has focused on improving the economics and nutrition by changing the raw materials and fermentation methods. It was found that most of the soy sauce produced in Brazil contained <20% of soybean and used corn as the raw material, which is cheaper (Morais, Pellegrinetti, Sturion, Sattolo, & Martinelli, 2018). Similarly, pretreatment of raw materials can also affect the quality of fermented soy sauce. Chou and Ling (1998) found that the contents of total nitrogen, amino nitrogen, free amino acids, reducing sugars, and the protein utilization rate of the soy sauce prepared using an extrusion pretreatment of raw soybeans were higher than those using traditional raw materials. Park and Park (2016) used halophilic bacteria to ferment defatted soy flour (DSF) without adding dried fermented soybeans or *meju* (soybean koji) to prepare soy sauce and found that the quality of the soy sauce directly fermented using DSF was better than that prepared using *meju*.

Walnuts are widely distributed throughout the world, and China is one of the major producing areas (Gao, Jin, Liu, Jin, & Wang, 2018). The increasing demand for walnut lipids has led to a large number of by‐products: Defatted walnut meal contains more than 40% proteins. Currently, it is mainly used for animal feed, and its low value offers opportunities to develop human foods (Gu et al., 2015). Chen, Zhao, et al. (2015) and Chen, Feng, Cui, Zhao, and Zhao (2015) studied the functional activities of walnut meal hydrolysates, which showed a stronger hydroxyl scavenging ability and oxygen free radical absorption capacity compared with glutathione (GSH) and cerebrolysin. Glutathione exists in almost every cell of the human body, and it has both antioxidant and detoxifying effects. Cerebrolysin has protective effects against cell degeneration (Chen, Zhao, et al., 2015; Chen, Feng, et al., 2015). Liang, Chen, Cao, and Zhao (2017) identified 12 polyphenols in cold pressed defatted walnut powder (DWP) extract and studied the potential of DWP to improve glucose and lipid metabolism in mice. Their results showed that the application of DWP inhibited mice weight gain and fat accumulation. Most studies on walnut meal focused on the functional analysis of its hydrolysates (Feng et al., 2019). However, research on fermented walnut meal is limited. Therefore, the objective of this paper is to determine the optimal conditions for the fermentation of walnut meal to produce a soy sauce‐like material, measure its basic composition, and compare it with commercial soy sauces. This study accessed the walnut soy sauce was extracted with different solvents to determine which extract had the best antioxidative stability and ACE inhibitory activity. These fractions were also characterized in terms of possible compounds contributing to these two activities. The findings would be beneficial for utilizing the walnut meal after oil extraction as a raw material to prepare soy sauce.

## 2 MATERIALS AND METHODS

### 2.1 Materials

Walnut meal (WM, cold pressed) was obtained from Hebei JingPin Fruit Co., Ltd. Wheat bran and three different commercial soy sauces were provided by Haitian Co., Ltd. *Aspergillus oryzae* (3.042) was obtained from the Microbiology Laboratory of Beijing Forestry University (Beijing, China). All chemical reagents were analytical grade and purchased from Tianjin Yongda Chemical Reagent Co., Ltd.

### 2.2 Preparation of koji

Walnut meal powder (~3,000 g) was soaked in distilled water for 20 min, and its moisture content was measured using AOAC method (AOAC, 2000). Then, it was mixed with bran in a ratio of 1:1 (w/w), and the mixture was autoclaved at 121°C for 30 min. Subsequently, the mixture was inoculated with *A. oryzae* at the ratio of 1:100 (fungus:WM powder, w/w) in a sterile room. The inoculated substrate was incubated at 30°C for 32 hr and finally dried at 45°C for 24 hr to obtain the koji.

### 2.3 Preparation of walnut soy sauce (WSS)

The koji and brine (13% NaCl, w/w) were thoroughly mixed at different mass ratios (1:1–1:3) and fermented at different temperatures (32–48°C) for 2–14 days. Each mixture was then centrifuged (HC‐2518R, Zonkla Scientific Instrument Co., Ltd.) at 1,484 *g* for 10 min at 25°C. The supernatant as raw WSS was transferred to brown bottles (250 ml) and stored at 4°C until analysis, a maximum of 8 weeks.

Based on the results from the single‐factor tests, response surface experiments (RSE) were used to determine the optimal conditions using three independent variables: mass ratio of brine:koji (A), fermentation temperature (B), and time (C).

### 2.4 Amino nitrogen content (ANC) assay

Amino nitrogen content of all samples was measured using the formol titration method (Dhanabalan et al., 2017). Briefly, diluted WWS (10 ml) was titrated with (0.05 mol/L NaOH) to a pH of 8.2. Then, 10 ml of neutral formaldehyde was mixed with treated samples and titrated to pH 9.2 with NaOH (0.05 mol/L).

### 2.5 Preparation of different solvent extracts of WSS

Walnut soy sauce (500 ml) was extracted successively with 100 ml of ethyl acetate, acetone, and n‐butanol for 30 min at 60°C, and the extracts were separated using a separatory funnel. The solvents were removed using a sequence of rotary evaporation at 60°C, vacuum drying (2 hr, 90°C), and freeze drying to obtain ethyl acetate extracts (EAE), acetone extracts (AE), n‐butanol extracts (BE), and water residue (WR). Then, the extract powders were dissolved in methanol and diluted to 50 ml for further testing.

### 2.6 Determination of free amino acids (FAA) in raw WSS and commercial soy sauces

The FAA was determined using a model A300 Amino Acid Auto‐Analyzer (MembraPure) according to Xie, Du, Shen, Wu, and Lin (2019) with minor modifications. Briefly, 5 g of the sample, 1 ml of phenol, and 10 ml of 6 mol/L HCl were added into a hydrolysis tube, and frozen (salt/ice = 1:3) for 5 min, then hydrolyzed at 110°C for 22 hr. The hydrolyzate was vacuum dried (45°C, 2 hr), and the final residue was dissolved in 1 ml of phosphate‐buffered saline (PBS, pH 2.2). The solution was filtered through a 0.22 μm microporous membrane and then injected into an analyzer to determine the amino acid composition.

### 2.7 Determination of active ingredients in solvent extracts

Total phenolic contents (TPC) of the WSS extracts were measured using the Folin–Ciocalteu method described by Hsiao, Gu, and Weng (2013). Gallic acid was used for the standard curves (0–0.01 mg/ml in distilled water), and the results were expressed as mg gallic acid equivalent (GAE)/g of solvent extract powder.

Total flavonoid content (TFC) was quantified using colorimetric assay (Granato, Santos, Maciel, & Nunes, 2016). A calibration curve of rutin (0–0.025 mg/ml in distilled water) was used, and results were expressed as mg of rutin equivalent (RE)/g of solvent extract powder.

Reducing sugar content (RSC) was determined using the 3,5‐dinitrosalicylic acid (DNS) method (Miller, 1959). A glucose calibration curve (0–1.8 mg/ml in distilled water) was used, and results were expressed as glucose equivalents/g of extract powder.

The polypeptide content (PC) was determined using CNDS (Chinese National Drug Standard, 2000). The bovine serum albumin was used to prepare a calibration curve (0–0.25 mg/ml in distilled water) for quantitative analysis. Results were expressed as mg of bovine serum albumin equivalents (BSE)/g of solvent extract powder.

### 2.8 Antioxidant capacity of different solvent extracts

DPPH (2,2‐diphenyl‐1‐picrylhydrazyl) scavenging activity was determined according to the method described by Smith, Reeves, Dage, and Schnettler (1987). The absorbance of the sample solution was measured using a spectrophotometer (L6, Yidian Analytical Instrument Co.) at 517 nm. Ascorbic acid (V_C_) and butylated hydroxyl toluene (BHT) were used as reference standards. All tests were done in triplicate, and the results were averaged.

Hydroxyl radicals (•OH) scavenging ability was determined according to the method of Li, Chen, Wang, Ji, and Wu (2007) and Li, Wei, White, and Beta (2007). The absorbance was measured at 510 nm. V_C_ and BHT were used as references. All tests were done in triplicate, and the results were averaged.

Superoxide radical (O_2_‐•) scavenging ability was determined using the method of Xie et al. (2016), and the absorbance of the sample solution was measured at 420 nm. V_C_ and BHT were used as reference standards. All tests were done in triplicate, and the results were averaged.

Reducing power was analyzed using the method described by Oyaizu (1986). The absorbance of the solution was determined at 700 nm. V_C_ and BHT were used as references. Absorbance of the reaction mixture represented the total reducing power. All tests were done in triplicate and the results were averaged.

### 2.9 ACE inhibitory activity of different solvent extracts

#### 2.9.1 Determination of reaction time

The reaction time was determined according to the method reported by Vermeirssen, Camp, and Verstraete (2002). An 80 μl of sample and 40 μl of ACE were mixed at 37°C in water bath for 10 min. Then, 200 μl N‐hippuryl‐his‐leutetrahydrate (HHL, 5 mol/L) solutions were added. The absorbance of the hippuric acid (Hip) was measured at 228 nm at various times, and the time to reach the maximum absorbance was obtained.

#### 2.9.2 Development of a method for determining ACE inhibition rate

The ACE inhibition rate was determined using HPLC. A Shimadzu LC‐2010A HT system (Shimadzu) equipped with VP‐ODS C_18_ column and SPD‐M20A diode array detector with detection wavelength of 283 nm was used. The mobile phase consisted of methanol and water (3:7, v/v,) at a flow rate of 1.0 ml/min and a column temperature of 30°C. Sample solution, ACE, and HHL were prepared using BBS (0.1mol/L, pH 8.3) containing 0.3 mol/L KCl as a solvent. Sample solution (40 μl) was transferred to a test tube, and 20 μl of ACE and 100 μl of HHL (5 mol/L) were added. The mixture was incubated for 1 hr at 37°C, and HCl (1 mol/L) was used to stop the reaction. The treated sample solution (10 μl) was filtered through a 0.45 μm microporous membrane and then injected into the HPLC to determine the amount of Hip. The ACE scavenging capacity was determined as follows.

$$\text{Inhibition}\;\left(\%\right)=\left(A_{0}-A_{1}\right)/A_{1}\times 100\%$$

where *A*_0_ is the peak area of Hip in the blank group and *A*_1_ is the peak area of Hip of the sample. Peak areas were determined by the software that came with the instrument.

### 2.10 Statistical analysis

All experiments were done in triplicate, and the data were analyzed using the Statistical Package for the Social Sciences (SPSS) Version 21.0 (SPSS Inc). The significant differences among means were tested using ANOVA followed by Duncan's test (*p* < .05).

## 3 RESULTS AND DISCUSSION

### 3.1 Effect of different process conditions on amino nitrogen content (ANC) of walnut soy sauce (WSS)

**Figure 1:**
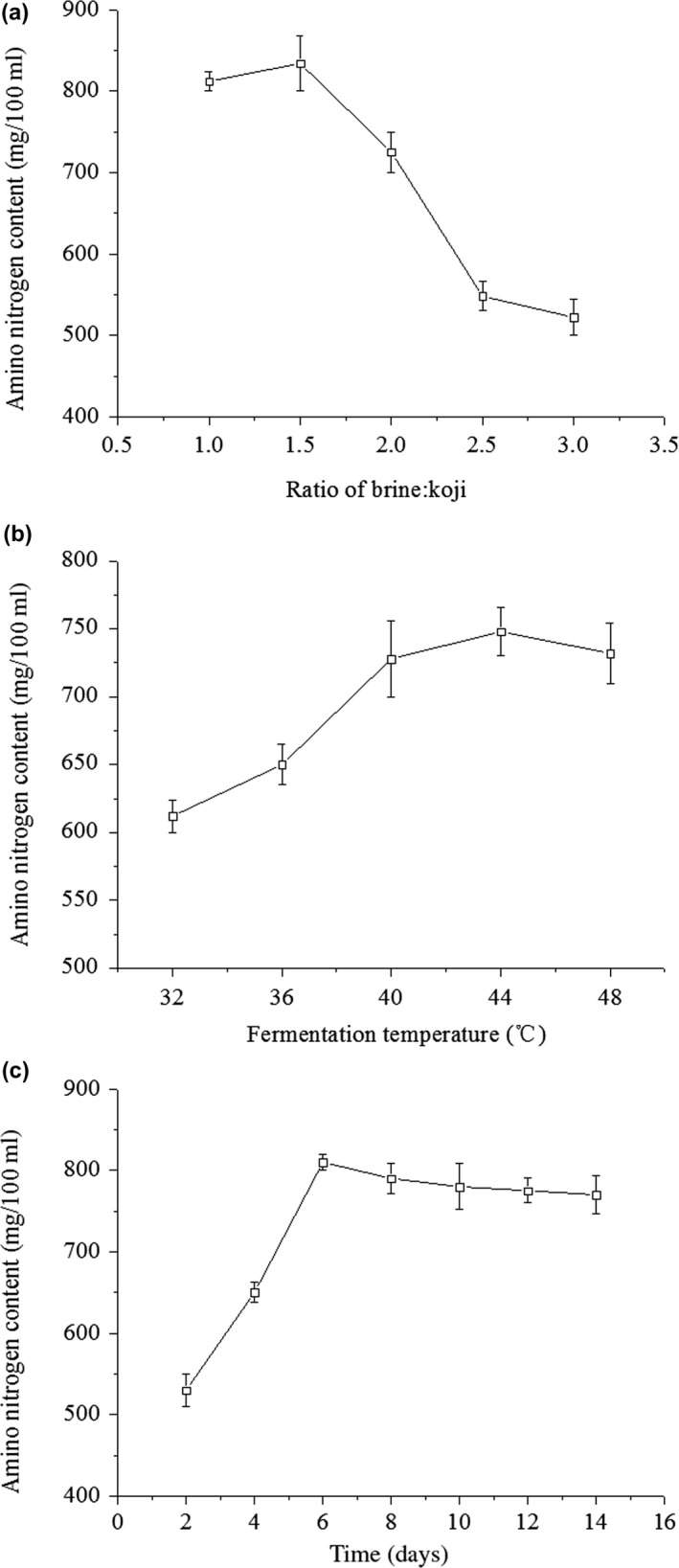
Effects of the ratio of brine:koji (a), fermentation temperature (b), time (c) on amino nitrogen content of walnut soy sauce

Amino nitrogen is one of the most necessary indicators for evaluating the quality of soy sauce products, and it has a great contribution to the nutritional value and sensory characteristics of soy sauce (Nakahara, Yamaguchi, & Uchida, 2012). The presence of amino nitrogen in soy sauce can be explained by the action of proteases and peptidases in the fermentation system, which hydrolyzed the protein of the raw material into peptides, amino acids, and small fragments of ammonia (Chen, Zhao, et al., 2015; Chen, Feng, et al., 2015). Amino nitrogen content varied with the different mass ratios of brine:koji (Figure 1a), and the maximum value was obtained at 1.5:1 of brine to koji. However, it decreased significantly from 834 to 544 mg/100 ml (*p* < .05) when the mass ratio of brine to koji increased from 1.5 to 3. This may be due to enzymatic denaturation caused by high brine content, and the spatial structure of the enzyme was changed, with low activity of proteases and peptidases. Besides, the decrease metabolism of microorganisms especially molds and yeasts, the enzyme activity was reduced and prolonged the fermentation time, thereby reduced protein decomposition and consequently exhibited low ANC (Wu, Kan, Siow, & Palniandy, 2010). Figure 1b shows that the ANC of WSS increased gradually as the temperature increased, and the highest value was obtained when the fermentation temperature was 44°C. Subsequently, the ANC decreased with the increasing temperature, which can be explained by the inactivation of the enzyme. Peptidase responsible for degrading the low molecular weight bioactive peptide was inactivated during the high temperature (~45°C) fermentation; thus, the amino nitrogen formed by the hydrolysis would be likely to remain in the low‐temperature fermentation (Nakahara et al., 2012). Amino nitrogen content increased when the fermentation time was extended from 2 to 6 days (Figure 1c), and there was no significant difference (*p* ≥ .05) as time increased to 14 days. This suggested the reaction was completed at 6 days, which represents the reaction time of peptidase degraded peptides into amino acids (Nakahara et al., 2010). Therefore, the fermentation conditions selected for the RSE were as ratio of brine:koji of 1–2, fermentation temperatures of 40–48°C, and times of 5–7 days.

### 3.2 Optimization of walnut soy sauce (WSS) fermentation conditions using response surface experiments (RSE)

Based on the results of single‐factor tests, the effects of parameters were studied to obtain optimized experimental conditions for the preparation of WSS, and the results of Box–Behnken design (BBD) are shown in Table 1. The following formula shows the ANC (Y) as a function of ratio of brine:koji (A), fermentation temperature (B), and time (C)by applying multiple regression analyses to experimental data.

$$Y=0.84+0.0\text{55A}+0.0\text{44B}-0.0\text{25C}+0.0\text{14AB}+0.00\text{6 AC}+0.0\text{51BC}-0.0{\text{96A}}^{2}-0.0{\text{61B}}^{2}-0.0{\text{4C}}^{2}.$$

**Table 1:** Experimental data for amino acid content of walnut soy sauce using the Box–Behnken design

No.	Coded levels of variables						Amino nitrogen content (mg/100 ml)
	Coded			Real			
	A	B	C	A	B	C	
1	−1	−1	0	1	40	6	591
2	−1	0	−1	1	44	5	701
3	−1	0	1	1	44	7	622
4	−1	1	0	1	48	6	630
5	0	−1	−1	1.5	40	5	749
6	0	−1	1	1.5	40	7	614
7	0	1	−1	1.5	48	5	757
8	0	1	1	1.5	48	7	824
9	1	−1	0	2	40	6	701.
10	1	0	−1	2	44	5	767
11	1	0	1	2	44	7	713
12	1	1	0	2	48	6	798
13	0	0	0	1.5	44	6	831
14	0	0	0	1.5	44	6	830
15	0	0	0	1.5	44	6	868
16	0	0	0	1.5	44	6	835
17	0	0	0	1.5	44	6	838

The adequacy and significance of the ANC of WSS is shown in Table 2. A very low *p*‐value (*p* < .0001) and the calculated *F*‐value (42.83) showed that the model was highly significant. In addition, the lack of fit was not significant (1.07), and the value of *R* ^2^ was .9674, which also indicated that the model fitted well with the actual test. The ANOVA showed that the BC, B^2^, and C^2^ terms had an extremely significant effect on ANC (*p* < .01), while AB and AC were nonsignificant (*p* ≥ .05). These results were in accordance with Hoang et al. (2016), and temperature–time interaction and both of quadratic terms of temperature and time exhibited negative effect on ANC. The optimum values of the tested variables from the model were as follows: ratio of brine:koji, 1.65:1; fermentation temperature, 45.5°C; time, 5.95 days, and the highest ANC was predicted to be 855 mg/100 ml.

**Table 2:** Regression equation analysis of variance

Source	Degree of freedom	Sum of square	Mean square	*F*‐value	*p*‐value
A	1	0.024	0.024	42.83	.0002
B	1	0.016	0.016	28.07	.0007
C	1	5.151 × 10^−3^	5.151 × 10^−3^	9.28	.0159
AB	1	8.410 × 10^−4^	8.410 × 10^−4^	1.52	.2532
AC	1	1.440 × 10^−4^	1.440 × 10^−4^	0.26	.6242
BC	1	0.010	0.010	18.57	.0026
A^2^	1	0.041	0.041	73.18	<.0001
B^2^	1	0.016	0.016	28.99	.0007
C^2^	1	7.055 × 10^−3^	7.055 × 10^−3^	12.72	.0073
Model	9	0.13	0.015	26.34	<.0001
Residual	8	4.439 × 10^−3^	5.548 × 10^−3^		
Lack of Fit	3	3.231 × 10^−3^	1.077 × 10^−3^	4.46	.0705
Pure Error	5	1.207 × 10^−3^	2.415 × 10^−4^		
Cor Total	17	0.14			

Validation test was done to verify the adequacy of the proposed model at a ratio of brine:koji, 1.7:1; fermentation temperature, 45°C; and time, 6 days. Using these conditions, the actual ANC of the WSS was 836 mg/100 ml, which was slightly lower than the predicted value, indicating that the model could be verified.

### 3.3 Free fatty acids (FAA) of walnut soy sauce (WSS) and the commercial soy sauces

During the fermentation preparation of soy sauce, the proteins in the raw material were degraded into peptides by endoprotease and then further degraded into FAA by exoprotease (peptidase) (Nakahara et al., 2012). All four samples have similar amino acid content including all of the essential amino acids as shown in Table 3. The essential amino acid content in WSS was 39.8% of the total amino acid content, and the nonessential amino acid content was 66.1%, which is close to the WHO/FAO recommended standard (Proll, 2010). Among FAA of the three commercial soy sauces, glutamic acid, lysine, isoleucine, and leucine were dominant. Free amino acids distribution was consistent with the characteristics of Chinese traditional soy sauce described by Cui, Zheng, Wu, and Zhou (2014). However, aspartic acid, glutamic acid, leucine, and arginine were dominant in WSS, which may be due to the difference in the preparation process of soy sauce and the raw materials used in fermentation. Amino nitrogen content is related to free amino acid content (Lu et al., 2009). Preparation process such as fermentation temperature and time will affect the content of amino nitrogen and then the content of free amino acids. Moreover, the arginine content of walnut protein was higher than that of soybean. Free amino acids was important for the flavor of soy sauce, and it can be divided into sweet, bitter, umami, and tasteless amino acids according to their taste (Lioe et al., 2004; Schoenberger, Krottenthaler, & Back, 2002). Umami FAA of Glu and Asp in WSS were significantly higher (*p* < .05) contents than those in commercial soy sauces, which may lead to WSS have more intense flavor.

**Table 3:** Free amino acid composition of walnut soy sauce and three commercial soy sauces

Amino acids	Taste	Soy sauces (mg/100g)			
		Walnut	A	B	C
Aspartic	Umami	271.4 ± 23.8^a^	116.6 ± 19.0^c^	124.0 ± 34.4^c^	212.4 ± 24.5^b^
Glutamic	Umami	432.8 ± 44.0^a^	233.8 ± 4.5^b^	351.1 ± 3.4^a^	278.5 ± 7.4^b^
Content of umami					
FAAs		704.2^a^	350.4^c^	475.1^b^	490.9^b^
Serine	Sweet	175.7 ± 7.1^b^	173.0 ± 8.4^b^	261.9 ± 21.3^a^	172.9 ± 11.7^b^
Glycine	Sweet	142.1 ± 1.3^b^	86.9 ± 3.8^c^	160.1 ± 1.2^a^	162.7 ± 1.2^a^
Threonine†	Sweet	166.2 ± 18.3^a^	118.9 ± 2.5^c^	175.3 ± 18.3^a^	142.5 ± 3.3^b^
Lysine†	Sweet/bitter	165.9 ± 2.7^c^	161.0 ± 6.7^c^	324.4 ± 16.2^a^	268.4 ± 3.8^b^
Proline	Sweet/bitter	95.9 ± 4.8^d^	162.9 ± 15.5^c^	284.4 ± 3.3^a^	255.5 ± 5.9^b^
Alanine	Sweet	181.1 ± 26.8^c^	173.2 ± 17.1^c^	283.2 ± 6.6^a^	262.6 ± 28.5^b^
Content of sweet					
FAAs		926.9^c^	873.9^d^	1,489.3^a^	1,264.6^b^
Arginine	Bitter	377.7 ± 22.1^a^	98.1 ± 0.5^b^	63.6 ± 3.6^c^	50.1 ± 1.8^c^
Tyrosine	Bitter	91.9 ± 4.8^b^	115.1 ± 4.7^a^	65.5 ± 5.9^c^	59.1 ± 0. 5^c^
Histidine	Bitter	92.7 ± 1.6^a^	54.6 ± 3.8^b^	73.7 ± 1.6^a^	75.7 ± 1.6^a^
Valine†	Bitter	211.6 ± 27.4^c^	192.9 ± 3.5^d^	328.2 ± 3.4^a^	272.1 ± 12.6^b^
Methionine†	Bitter	55.4 ± 1.3^b^	43.2 ± 5.1^c^	83.7 ± 0.2^a^	74.4 ± 4.2^a^
Phenylalanine†	Bitter	178.9 ± 3.9^c^	153.8 ± 2.9^d^	289.1 ± 2.2^a^	256.8 ± 15.9^b^
Isoleucine†	Bitter	186.4 ± 23.5^b^	174.0 ± 7.4^b^	307.0 ± 26.1^a^	289.9 ± 15.9^a^
Leucine†	Bitter	288.0 ± 15.2^c^	293.2 ± 6.6^c^	503.7 ± 18.2^a^	412.9 ± 20.5^b^
Content of bitter					
FAAs		1,482.6^b^	1,124.9^c^	1,714.5^a^	1,491.0^b^
Cysteine	ND	32.8 ± 1.9^a^	15.7 ± 1.5^d^	23.6 ± 4.1^b^	20.9 ± 1.8^c^
Content of total					
FAAs		3,146.5^b^	2,364.9^c^	3,702.5^a^	3,267.4^b^
Contents of essential					
FAAs		1,252.4^c^	1,137.0^d^	2,011.4^a^	1,717.0^b^
E/T (%)		39.8^d^	48.1^c^	54.3^a^	52.6^b^
E/N (%)		66.1^d^	92.6^c^	119.1^a^	110.9^b^

Note

Data are expressed as the mean ± *SD* (*n* = 3).

Different small letters indicate significant difference in a row (*p* < .05).

E/T (%): Ratio of essential amino acid content to total amino acid content.

E/N (%): Ratio of essential amino acid content to nonessential amino acid content.

Abbreviation: ND, not detected.

^†^ Essential free fatty acids (FAAs).

### 3.4 Active ingredients in solvent extracts

The composition and content of the extracts obtained with different solvents were expected to be different (Li, Chen, et al., 2007; Li, Wei, et al., 2007). It can be seen from Table 4 that WR gives the highest yield and EA the lowest. All the WSS extracts contained phenolic compounds and flavonoids, and their contents varied with solvent. Besides phenolic compounds and flavonoids, water residue as expected also contained some polypeptides and reducing sugars (Table 3) which were not found in the other solvents. These small amounts of peptides and reducing sugars are reasonable since the other three solvents used in this study were not suitable for extracting these fractions from the sample solution, while using pure water as the extraction solvent, above 100°C and ultrasonic‐assisted extractions, can effectively extract sugars and proteins (Mussatto & Roberto, 2006). The content of total phenols in WSS extracts in ethyl acetate was 20‐fold higher than that in water. This may be due to the phenolic compounds are not effectively extracted by water, as these compounds are generally more soluble in organic solvents less polar than water (Kim & Lee, 2003). Acetone/water mixtures (50%–95%, v/v) have been reported to be one of the most effective solvents for extraction of phenols from different natural sources, especially from protein matrices, since this mixture is able to break polyphenol–protein complexes (Kallithraka, Garcia‐Viguera, Bridle, & Bakker, 2010). However, acetone alone extracted less phenol than EA.

**Table 4:** Indices of different solvent extracts of walnut soy sauce

Samples	Indices				
	Yield (mg/ml WWS)	TPC(mg GAE/g*)	TFC(mg RE/g*)	RSC(mg/g*)	PC(mg BSE/g*)
EAE	2.7 ± 0.1^d^	20 ± 1^a^	24 ± 1^a^	ND	ND
AE	8.2 ± 0.2^c^	18.7 ± 0.4^b^	23 ± 1^b^	ND	ND
BE	10.4 ± 0.3^b^	19 ± 1^b^	22 ± 1^c^	ND	ND
WR	15.1 ± 0.2^a^	0.98 ± 0.17^c^	0.02 ± 0.00^d^	214 ± 4^a^	135 ± 1^a^

Note

Data are expressed as the mean ± *SD* (*n* = 3).

Different small letters indicate significant difference in a column (*p* < .05).

Abbreviation: ND, not detected.

Per gram of solvent extract.

### 3.5 Antioxidant activity of solvent extracts

#### 3.5.1 DPPH scavenging activity

**Figure 2:**
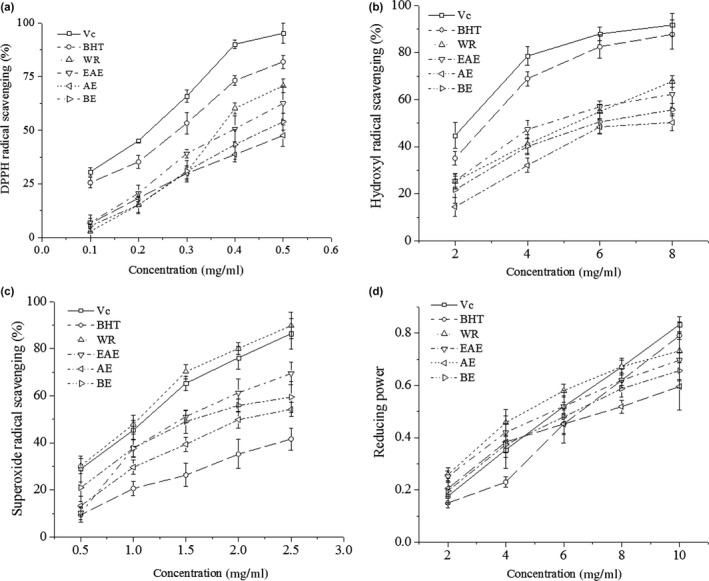
In vitro antioxidant activities of V_C_, BHT, ethyl acetate extract (EAE), acetone extract (AE), n‐butanol extract (BE), and water residue (WR): (a) DPPH radical scavenging activity; (b) hydroxyl radicals scavenging activity; (c) superoxide removal activity; (d) reducing power

2,2‐Diphenyl‐1‐picrylhydrazyl scavenging activities of all the samples had concentration dependency and showed higher scavenging ability with increasing concentration as shown in Figure 2a. At lower concentrations (<0.3 mg/ml), the AE showed higher DPPH activity than BE and WR. However, AE showed a lower value than EAE at high or low concentrations, which meant that the DPPH scavenging activity improved with the concentration of total phenols and flavonoids increased. These results were consistent with those reported by Meneses, Martins, Teixeira, and Mussatto (2013). Water residue showed the highest DPPH scavenging activity, which was above 0.35 mg/ml. This may be due to the high concentration of reducing sugars and peptides in WR with stronger DPPH free radical scavenging capacity, but still lower than V_C_ and BHT.

#### 3.5.2 Hydroxyl radicals scavenging ability assay

Hydroxyl radicals are not only an active agent for the peroxidation of lipids, but also the most active free radicals in biological tissues, especially affecting proteins (Asghar, Abdul Raman, & Wan Daud, 2016). High activity of hydroxyl radicals might affect the oxidative stability of WSS. As shown in Figure 2b, the difference in hydroxyl radical scavenging capacity was not significant (*p* ≥ .05) in all samples at a concentration of 6.0 mg/ml. However, AE showed the lowest hydroxyl radicals scavenging value at all concentrations.

#### 3.5.3 O_2_‐chelating activity

Figure 2c shows that there is a qualitatively positive relationship between the superoxide anion removal rate and the concentration of each solution. n‐Butanol extracts showed higher O_2_‐chelating activity than EAE and AE. However, the removal of superoxide with WR at the same concentration was higher than that of the other extracts, even slightly higher than that of V_C_. The WR may have contained a large number of active peptides containing more aromatic amino acids (Li, Jiang, Zhang, Mu, & Liu, 2008). All the solvent extracts had higher removal rates than the synthetic antioxidant BHT, probably because of the poor solubility of BHT in this system. In comparison, V_C_ which is water‐soluble did have a good dose–effect relationship within the studied concentration range.

#### 3.5.4 Reducing power

Reducing power is an important indicator of the antioxidant capacity of a substance, and there may be a correlation between reducing power and antioxidant activity (Wang, Gao, Zhou, Cai, & Yao, 2008). Compared with EAE and other extracts, WR showed significantly higher reducing power (*p* < .05), but lower than that of V_C_ at concentrations over 8 mg/ml (Figure 2d). No significant difference (*p* ≥ .05) in the reducing power between EAE and BE was observed, but both were higher than that of AE.

### 3.6 ACE inhibitory activity

#### 3.6.1 Development of the ACE inhibitory activity method

**Figure 3:**
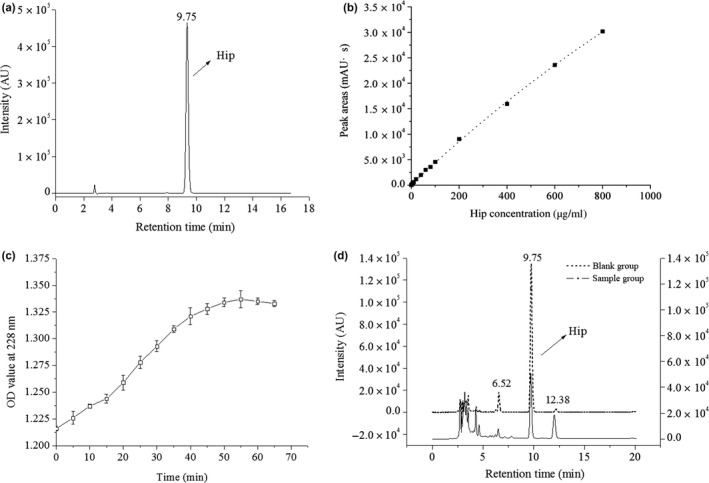
Method for determining ACE inhibitory activity of walnut soy sauce: (a) the HPLC chromatogram of hippuric acid standard substance; (b) standard curve of hippuric acid concentration versus peak area; (c) hippuric acid absorbance (228nm) with reaction time of ACE and HHL; (d) the HPLC chromatogram of blank group and sample group

N‐Hippuryl‐His‐Leutetrahydrate is decomposed by ACE to produce Hip, which has a characteristic absorption peak at 228 nm. The amount of Hip produced will be reduced when ACE activity is inhibited. A HPLC scan of Hip is shown in Figure 3a, which indicates that the Hip chromatographic peaks appeared at a reaction time of 9.75 min. Figure 3b shows that there is a linear relationship between the Hip concentration and the peak area. In addition, as shown in Figure 3b, the minimum detection limit of Hip is 0.5 μg/mL, and the standard regression curve of concentration and peak area is *y* = 38.31*x* + 288.0, *R* ^2^ = .9979. The linear relationship between the concentration of Hip and of the peak area shows that the HPLC response quantitatively measures Hip. It can be seen from Figure 3c that the Hip increases gradually over time during the reaction of ACE with HHL, then decreases slowly after reaching a maximum at 55 min, and finally remains stable at 60 min. So, the reaction time for determining the ACE inhibitory activity in this experiment was determined to be 1 hr (Vermeirssen et al., 2002). Figure 3d shows the change of chromatogram before and after the addition of the sample solution, and the peak area of Hip after the addition of the sample solution significantly reduced. Therefore, the Hip content generated was reduced, and the ACE activity was suppressed.

#### 3.6.2 ACE inhibitory activity of different solvent extracts

**Figure 4:**
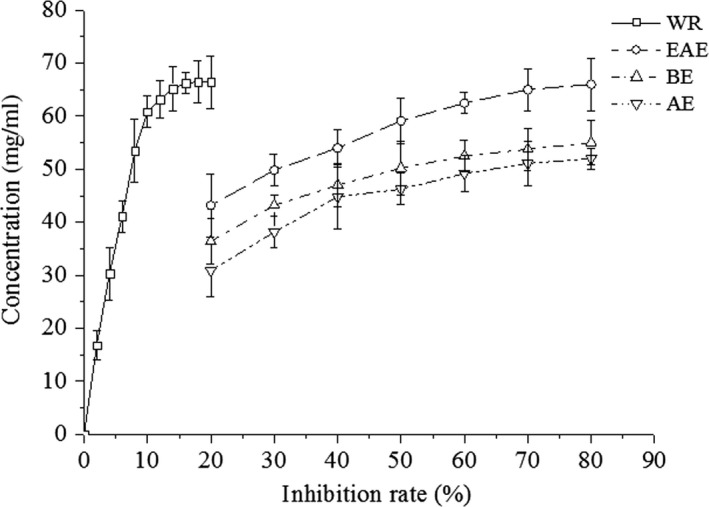
ACE inhibition rate of different solvent extracts

The ACE inhibitory activity of extracts from different solvents of WSS is shown in Figure 4. All four extracts have in vitro ACE inhibitory activity, and its inhibition rate is positively correlated with extract concentration, as also reported by Sheih, Fang, and Wu (2009). The ACE inhibition rate of WR was around 66.5% at 20 mg/ml, which was the highest inhibitory activity in all extracts, and the other samples at various concentrations ranged from 51.9% to 65.8%. Moreover, the ACE inhibitory activity of these extracts tended to level off at concentrations close to 80mg/ml. In general, water extracts had the best ACE inhibitory activity (IC_50_ = 8.20 mg/ml) compared to EAE (IC_50_ = 31.5 mg/ml), AE (IC_50_ = 57.5 mg/ml), and BE (IC_50_ = 77.2 mg/ml), which also indicated that the substance(s) with antihypertensive activity was mainly present in the WR. These results may be due to the polypeptides of the walnut protein. However, the other three extracts also had ACE inhibitory activity, probably because of their phenolic substances, but the effect of these compounds was much lower than that of peptides. The ACE inhibitory activity of the extract indicates that WSS has potential for lowering blood pressure, but the effect is much lower than that of the high‐efficient antihypertensive drug captopril (not shown in the figure), and further research to enhance this inhibitory activity is necessary.

## 4 CONCLUSION

In general, this study indicates the feasibility of using walnut meal to prepare soy sauce. Walnut soy sauce with high amino nitrogen content was obtained by fermentation of walnut meal, and the conditions for the preparation of walnut soy sauce were optimized using response surface experiments. When the ratio of brine to Koji was 1:7, the amino nitrogen content in walnut soy sauce fermented at 45°C for 6 days was the highest. Also, all the results of amino acid analyzer showed that walnut soy sauce has higher umami amino acid content than other three commercial soy sauces. Basic composition analysis of walnut soy sauce extracts showed that the total phenolic and flavonoid content of ethyl acetate extracts were highest. However, the reducing sugars and polypeptide content of water residue was the highest, which gave it the strongest antioxidant capacity and ACE inhibitory activity. Based on the above results, the walnut soy sauce has antioxidant and ACE inhibitory activity. This product may replace traditional soy sauce to a certain extent and improve the use of walnut meal. Furthermore, the detailed composition and commercial value of walnut soy sauce remains to be studied.

## CONFLICT OF INTEREST

The authors have no conflicts of interest with respect to this manuscript.

## ETHICAL APPROVAL

This study does not involve any human or animal testing.
